# Can the Concepts of Energy and Psychological Energy Enrich Our Understanding of Psychosocial Adaptation to Traumatic Experiences, Chronic Illnesses and Disabilities?

**DOI:** 10.3389/fpsyg.2022.768664

**Published:** 2022-03-03

**Authors:** Hanoch Livneh

**Affiliations:** Clinical Rehabilitation Program, Department of Counselor Education, Portland State University, Portland, OR, United States

**Keywords:** energy, psychological energy, psychosocial adaptation, chronic illness, disability

## Abstract

The aim of this paper is to familiarize the reader with the concept of psychological energy (PE), and the role it plays in deepening our understanding of psychosocial adaptation to traumatic life events and, more pointedly, the onset of chronic illness and disability (CID). In order to implement this aim, the following steps were undertaken: First, a brief historical review of the nature of energy, force and action, as traditionally conceived in the field of physics, is provided. Second, an overview of PE is presented, with a shared emphasis on both its historical underpinnings and its present conceptualizations in the fields of social, health and rehabilitation psychology. Particular emphasis is placed upon applications of PE in the domains of adaptation to stress, trauma and CID onset. Third, reviewed are measuring instruments that have been traditionally applied to the assessment of the nature, content and magnitude of PE and its dynamics. Finally, new perspectives are offered on the dimensional structure, processes and dynamics, assumed to undergird PE, its underlying conceptual similarities to physical energy, and its potential and deeper link to the process of psychosocial adaptation in the aftermath of experiencing trauma and CID.

## Introduction

Energy may be one of the most overlooked concepts in the fields of rehabilitation and trauma psychology, and, more specifically, in understanding psychosocial adaptation to chronic illness and disability (CID). Indeed, extensive literature reviews of such sources as Google Scholar, Web of Science, Academic Search Premier, Medline, and others, have yielded only scarce references to the role that energy plays in understanding adaptation to crisis, trauma, and more pointedly, sudden onset CID. The concept of psychological energy (PE), or in its more commonly used terms, such as motivation, vitality, drive, has also been mostly underappreciated and generated only a moderate degree of interest in social, health, and clinical psychology (Baumeister and Vohs, 2007; Ryan and Deci, 2008). PE typically refers to the dynamics that empower human motivation and vitality. It also implies a direction-oriented force that fuels the pursuit and achievement of life goals and plans (Lewin, 1951; Wright, 1987; Baumeister, 2002). PE may be regarded as a second order derivative of physical energy, biological energy being a first order derivative. Indeed, a temporal perspective on the concept of energy suggests that energy (and its equivalent, matter) was created during the “big bang” era (Carroll, 2016). It was only when organismic life first evolved that biochemical energy made its first appearance, followed by the emergence of rudimentary PE when the first humanoids walked the face of planet earth (Greene, 2020). Finally, PE, with modified manifestations of energy, gradually emerged, spawned by the availability of more complex cognitive structures and the presence of self-consciousness (Carroll, 2016; Davies, 2019).

Physical energy, unlike PE, is a readily measurable construct, yielding to mathematically precise equations. PE, in contrast, has to be indirectly inferred from an organism’s intentions and behaviors and presents more complex measurement obstacles and a mathematically obscure picture. Physical energy and PE, however, also address different, and at times incompatible, domains of functioning as will be further explored later on in this paper. At its core, energy connotes a dynamic, yet conserved interaction between an object’s mass and its momentum (Greene, 2020). In the domain of biophysics, the essence of energy is depicted by the organism’s capacity to do work, that is, to obtain and process energy from its surroundings in order to stay alive (Davies, 1999). PE, in turn, paints a far richer picture, not the least of which is an emphasis upon the (personal) quality of its multileveled manifestations. Most significantly, what distinguishes PE from physical energy, is its capacity to avoid any rigid conservation limits that are imposed on physical energy, that is, its inherent flexible outreach across both space and time. PE, therefore, is a uniquely malleable concept, allowing it to be both depleted and enhanced through the deployment of various biochemical, social and psychological means (Hobfoll, 1989; Deci and Ryan, 2000; Baumeister, 2002).

One of the unique arenas where the nature, structure and dynamics of PE can be studied is that of life with CID, since the presence of CID exerts a heavy toll on daily activities, often depleting energy resources (Barker et al., 1953/1977; Lazarus and Folkman, 1984; Baumeister, 2002). Despite having been seldom examined in the domains of disability studies and rehabilitation, the role of PE in elucidating psychosocial adaptation to CID, nevertheless, offers much promise. Indeed, a close scrutiny of the definition of adaptation to CID helps to better understand the role PE plays in fueling it. Adaptation to CID was defined as “an evolving, dynamic, general process through which the individual gradually approaches an optimal state of person-environment congruence manifested by (1) active participation in social, vocational, and avocational pursuits; (2) successful negotiation of the physical environment; and (3) awareness of remaining strengths and assets as well as existing functional limitations “(Livneh and Antonak, 1997, p. 8). The focus on such terms as evolving, dynamic, active participation, and goal pursuit clearly speaks to the energy-infused process undergirding adaptation to CID. Furthermore, PE has been recognized by prominent scholars, for a century (Freud, 1923/1960; Lewin, 1935; Shontz, 1975; Lazarus, 1993), as the force field that permeates one’s life space, sustains mental and emotional experiences, and, as will be explored in this paper, determines to a large degree, the tapestry of psychological reactions and coping resources adopted following the onset of CID.

Numerous studies have repeatedly reported that people with CID cope with a multitude of daily hassles that emanate from the nature of their condition (e.g., mobility restrictions, pain), the surrounding physical environment (e.g., architectural barriers, limited opportunities, rigid medical regimens), and related psychosocial factors (e.g., stigmatizing attitudes, occupational discrimination, uncertainties such as prognosis and condition duration) (Smart, 2009; Corrigan, 2014). Their constant struggles, and triumphs, therefore, exert an immense toll on their energy resources and coping efforts, as well as extends across a wide range of life domains. Understanding the concept of PE within the context of life with CID could, then, serve to improve the quality of life (QOL) of people with CID.

In a similar manner, a clinically rich array of experimental and evidence-based assessments and interventions have been advanced by several of the PE models, and their applications to the field of adaptation to CID, could positively impact QOL among individuals coping with the aftermath of CID. PE-infused conceptual models, assessment procedures and therapeutic modalities, although mostly in earlier stages of development, nevertheless, offer opportunities for further exploration of the possible link between PE and the many facets of life with CID (Ryan and Deci, 2008; Gallo, 2009; Ntoumanis et al., 2021).

In this review paper, therefore, a multifocal approach aimed at examining the role that PE plays in elucidating the nature, structure and dynamics of psychosocial adaptation to trauma and CID is undertaken. To pursue this aim, first, a brief overview of the meaning, nature and pertinent laws of physical energy is presented. Second, a critical review of the available literature on theories of PE is provided, focusing on PE’s role in theories of personality, coping, stress, and trauma. Third, the role of PE in the context of life with CID is addressed. Fourth, approaches to the measurement of PE are outlined, briefly considering their psychometric soundness. Finally, perspectives on the potential role PE could play in the field of adaptation to CID, including suggestions on how its implementation within this field, might provide a better understanding of adaptation to CID are presented.

## Energy, Force, and Action

The term energy (in Greek, *Energeia* refers to “doing work,” or “within work”) can be traced back to Aristotle (384–322 BCE). However, it was only in the mid-16th century that the term was used to denote force or vigor of expression. The understanding of energy reached its modern interpretation when Thomas Young (circa 1802) first used the term energy to indicate it as being a product of the mass of a body into the square of its velocity (Smith, 1998). Energy^1^ is often used interchangeably with such terms as force, activity, action, strength, dynamism, motion, and momentum. It is regarded as the capacity of a body, or system, to do work, where work is specified by a given amount of force acting through distance. The acquisition of energy from the environment is also the process that organisms adopt in order to function, since the initiation and maintenance of life require removal of the organism, periodically, from equilibrium (Davies, 2019).

The concept of energy occupies a cardinal role in our understanding of the world, and in a wide range of physics theories. Indeed, the first two laws of thermodynamics directly address energy. The first law (“conservation of energy”) posits that energy (or mass energy) cannot be destroyed or created, in a closed system, but can be modified from one form to another. In a similar vein, the second law (“increase in entropy“) maintains that, in a closed system, entropy (a measure of the system’s level of disorder) cannot decrease, thus indicating how much energy is unavailable to the system to do its work (Krauss, 2017). Both of Einstein’s (1879--1955) theories, namely, the special and general theories of relativity, are inherently linked to energy.^2^ Indeed, in general relativity, energy in any of its forms, is the source of gravity and the initiator of the curvature of spacetime (Krauss, 2017).

Force^3^, derived from energy, refers to the power, strength, or amount of energy exerted, and is seen as the cause of motion or change. The concept of force played a major role in Newton’s (1643–1727) laws of motion, such that his first law implicates force as the product of mass and acceleration (F = MA). Finally, action, a derivative of the operation of force, has been incorporated more fully into the fields of both biology (viewed as function of the body or one of its parts) and psychology (viewed as an act of will or an organized activity to achieve a goal). It also plays a substantial role, as will be discussed later, in understanding and effecting adaptation to traumatic events and life crises, such as the onset of CID.

## Psychological Energy: Concepts and Theories

Unlike physics-linked energy, which is mostly defined by its magnitude, and measured by a unit of work termed (kilo)joules, PE is typically defined by four characteristics, namely, *magnitude* or valence (its strength), *direction* (physical energy, being a scalar, has no direction), *inner quality* (physical energy possesses no inherent quality or attribution of positiveness/negativeness), and *temporal extension* (in physics, energy, in a closed system, is conserved and, therefore, time-independent). A wide range of approaches to conceptualizing and examining PE has been offered in the psychological literature. Those deemed most influential are reviewed below.

### Freud and Psychoanalytic Theory

Historically, the term PE may be traced to the early years of the 20th century and the psychoanalytic theories of Sigmund Freud (1856–1939). Although several of Freud’s views on PE (e.g., the conflicting nature of psychic forces, the dynamic interaction among these forces) were predated by philosophers and scientists such as Johann Friedrich Herbart (1776–1841) and Carl Reichenbach (1788–1869), among others, his was the first comprehensive theory of the mind. In his *The Ego and the Id* (1923/1960), Freud, as part of his economic model of energy, developed the concept of drives and endowed them with displaceable and transformative quality of energy (drive energy; see also, Rappaport, 1960). He viewed PE as neutral in nature and as capable of being attached to various human impulses, most notably constructive (Eros, sexuality, life) and destructive (Thanatos, aggressiveness, death). Later, *in An Outline of Psychoanalysis*, he expanded his notion that energy fuels mental life, by arguing for the existence of two types of energy, mobile (free) and bound (attached) ones (Freud, 1940/1969). The former is linked to the id (the reservoir of primary, instinctual, unconscious processes), while the latter resides in the ego (the site of secondary, rational, conscious processes). The reservoir of this psychic energy was regarded as sublimated energy (desexualized) and was seen as residing in both the id and the ego and, therefore, acted to propel (cathect) behavior and imbue it with goal-directness and purpose. The adaptive energy resources available to the ego are quantitatively restricted and are, therefore, reduced when intra-psychic conflicts and repressive forces are present. When inner tension is built up, the person experiences displeasure, while discharge of tension is equated with pleasure (Pines, 1990). Freud was influenced by late 19th century and early 20th century physics perspective and, accordingly, viewed these psychic energies as obeying the principle of conservation of energy. They were, then, non-directional in nature (being scalars and not vectors) and their dynamic transformations, belied their drive origin and accounted for the “work” performed by the psychic force (Rappaport, 1960).

### Lewin and Dynamic Personality

A second early contributor to the field of PE is Kurt Lewin (1890–1947). Lewin’s energy model was influenced by Freud’s contributions and a conceptual similarity exists between Freud’s concepts of energy and drives and Lewin’s concepts of tension and forces (or valences), respectively (Rappaport, 1960). From the perspective of organismic economy, both models reflect phenomena that obey the known laws of physics (e.g., conservation of energy, entropy, principle of least action). In his dynamic, interactive and Gestalt-influenced theory, Lewin (1935) maintained that for psychological and behavioral processes to take place, work energy must be released. His somewhat rigid view of energy led him to conclude that “*one must therefore inquire of every psychical event whence the causal energies come*” (Lewin, 1935, p. 46). Although Lewin was a staunch supporter of using physics-based analogies, he adamantly argued that the concepts of energy, force and tension, representing psychical field forces, despite their embeddedness in the physical environment are, nevertheless, independent of it and maintain their own functional existence. Lewin was also quick to point out that the relationships between the amount of energy and the magnitude of forces, in both physics and psychology, is a non-linear one, such that weak forces could initiate large amounts of energy, and strong forces could result in small levels of energy, depending on the dynamics of the entire field. The concept of psychic forces was of prime importance in Lewin’s theory and was imbued with the power to influence and control the course of psychological processes (e.g., affective processes can only arise when strong forces, powered by intense psychic energies, are present and stay in motion). At the same time, the same forces that operate at both the inner (the person) and outer environments are also subject to change by the psychological process itself. All behaviors are fueled by the totality of these interdependent and co-occurring (Gestalt-determined) psychic forces (needs and goals) that comprise a dynamic equilibrium of one’s life space (Lewin, 1951).

In the larger context of the literature on biopsychological life space (Lewin, 1935, 1951; Shontz, 1975; Livneh et al., 2014), it could be argued that (life) energy reflects physical-like force fields that carve out a person’s life space. Furthermore, these force fields provide the valance that fuel LS with its goal-oriented, task-completing energy. In open systems, of which human functioning is a prime example, and as viewed through the lens of adaptation to trauma and CID, PE must now be rechanneled. Prior to experienced trauma or CID onset, PE focused on implementing and maintaining certain life schema, belief systems, and personal goals and plans. However, following these traumatic events it is forced to undertake new directions. In sum, according to Lewin’s theory, the course of psychological processes, and their embedding force fields, comprising the LS, can be envisioned as a complex, interactive and stochastic unfolding of affective and behavioral processes that are determined by: (a) the availability of PE, (b) the forces of the action process itself, and (c) the strength of the forces in the external field (Lewin, 1935; Rappaport, 1960).

### Selye and Stress Theory

A third historical figure whose contributions to PE left an indelible impact on this field is Hans Selye (1907–1982). In his adaptation to stress and physical health theory, Selye (1950, 1975) suggested that when an organism is exposed to a set of environmental stimuli (i.e., stressful events) whose intensity or quality exceeds that for which it has been adapted to, then the ensuing reaction generates what he referred to as *general adaptation syndrome* (GAS). Three distinct stages characterize GAS, namely, alarm reaction, resistance and exhaustion. The exhaustion stage signals loss of acquired adaptation. Since every organism is equipped with a finite and limited amount of “adaptational energy” (genetically constrained) and when this energy is fully consumed, the performance of adaptive life activities is no longer feasible. This restricted reservoir of adaptational energy may be exhausted by both physical and psychological demands. It can be consumed slowly and monotonously or be spent vigorously and unevenly during the course of a lifetime (Selye, 1975). However, when energy is depleted, both the psychic capacity to cope with stress and the body’s immunological system to ward off diseases are compromised. At that stage, it is equated with the GAS’s exhaustion stage and may lead to the individual’s ultimate death. Gorban et al. (2016) further concluded that according to Selye “adaptive energy is considered as an internal coordinate on the ‘dominant path’ in the model of adaptation” (p. 127). Motivational arousal is, therefore, critical to the understanding of Selye’s GAS. Indeed almost 70 years ago, Goldstone (1952) referred to adaptive energy as a “capital reserve of energy” and to death as a “bankruptcy of adaptive energy.”

### Contemporary Models of Psychological Energy

Several premises appear to undergird modern views on PE. They include: (a) energy, including PE, is of limited and depletable amount and, therefore, is exhaustible by both physical and psychological demands; (b) energy mobilization is linked to such processes as motivation, vigor, vitality and directed action; (c) motivational arousal is triggered by one’s needs, goals and plans; (d) the onset of stressful and traumatic experiences, employment of coping efforts (mostly active), and continuous self-control activities, all serve to drain energy resources; (e) when PE is exhausted, psychosocial functioning and physical survival are threatened; and (f) energy resources can be replenished (i.e., energy consumption) through activities, such as sleep, exercise, diet and social support.

In this section, six of the leading models, instructive to understanding the link between energy and PE, are briefly reviewed (see Table 1). In their *energization and motivation theory*, Brehm and Brehm (1981), Brehm et al. (1983), and Wright (1987) have posited three primary determinants that underlie energy mobilization, namely, the current state of the person (biological and psychosocial needs), the value of the presented incentive (positive or negative), and the expectancy of motive satisfaction. Motivation is associated with increased energy mobilization (its physiological component), with the magnitude of goal valence (its subjective component) and with the exerted effort, indicated by the speed, vigor and directedness of action (its behavioral component). Motivational arousal, or energization, therefore, should directly increase with one’s experienced need, and the value of the incentive or goal valence. The authors reasoned that if a motive (such as averting an environmental stressor) is easy to satisfy, or to cope with, or if an outcome is very difficult, or impossible, to attain, only little energy will be expended. On the other hand, if a motive (e.g., avoiding a stressor) is moderately difficult to cope with, then energy mobilization will be higher. Once an activity has been selected, the energy mobilized to fuel such an activity is proportional to the perceived difficulty of that activity. In medical and rehabilitation contexts, a negative event (e.g., an upcoming surgery, awaiting chemotherapy), should mobilize higher energy levels (as well as be perceived as more aversive) if coping efforts are difficult to employ, yet are justified and of probable success level. In contrast, the same negative medical event will yield lower energy investment if coping efforts are perceived to be easy or impossible to deploy, or are unwarranted (Wright, 1987). Empirical findings have supported the authors energy mobilization and motivation theory, including the claim that efforts are proportional to task difficulty when success is obtainable and justifiable, and that physiological (i.e., cardiovascular) reactivity, as a measure of energy mobilization, varies with the difficulty of coping efforts (Gendolla et al., 2012; Wright, 1996).

**TABLE 1:** Selected psychological energy theories and their main postulates.

PE-related theory	Contributing authors	Primary postulates
Energization and motivation theory	J. W. Brehm, S. S. Brehm, Wright	• Motivation is linked to increased energy mobilization • Energization (motivation) increases with needs, incentives and goal strength • Negative medical events mobilize higher energy levels when coping efforts are limited yet justified
Self-regulation theory	Baumeister	• Continuous self-control activities exhaust energy reservoir, thus constricting self-regulation • Energy/ego depletion is equated with reduced self-control • People possess limited or depletable energy and resource reservoir • Traumatic events require coping efforts, further depleting personal resources
Self-determination theory	Ryan, Deci	• Self-energy or vitality is a primary indicator of health and motivation • Vitality is a major link to resilience to stress and disease • Energy and vitality are enhanced when basic psychological needs are met • Autonomous and volitional self-regulation increase energy and vitality
“Moving forward with life” model	Greenglass	• Vitality is expressed as positive affect, vigor and “moving forward with life” • Vitality’s dimensions are closely associated with available social support, proactive coping, and independent functioning
Conservation of resources theory	Hobfoll	• People seek to obtain, retain and protect valuable resources • When valuable resources are threatened or lost stress is triggered • Four resource categories exist and they are: object, condition, personal and energy resources • Stressful events deplete resource pool and active coping modes, threatening physical and psychological functioning
Coping with Stress theory	Lazarus, Folkman	• Adaptive coping and energy level are linked to overall health status • People with CID possess less energy to expand on coping activities, especially under stressful situations • Appraisal of stress modalities is related to both energy valence and temporal orientation

The contributions of Baumeister (2002, 2003) to the understanding of PE must also be acknowledged. In his *self-regulation theory*, Baumeister et al. (1994) and Baumeister and Vohs (2007) argued that various behavioral problems (including some chronic diseases) are linked to self-regulation failure, in contrast to successful regulation activities that facilitate positive adaptation. Speared by self-control, which itself is of restricted temporal range, self-regulation is also of limited capacity. Therefore, engaging in continuous self-control activities gradually exhaust its reservoir of energy, and when depleted, further self-regulation is hampered (Hagger et al., 2010). This state of reduced strength of self-control was termed *ego depletion*. In his energy- or ego-depletion model, Baumeister (2002, 2003) posited that people possess a limited and depletable reservoir of resources and that this available energy, in turn, dictates personal efforts that regulate and control one’s internal states and processes, including emotions (affect regulation), cognitions (thought control), actions (task performance) and impulses (impulse control). Furthermore, Baumeister suggested that the centrality of energy resources extends beyond self-regulation and self-control to include a set of executive functions (e.g., choice, volition, suppressing thoughts and feelings, assuming responsibility). When the self’s energy resources, earmarked for self-regulation and volition, have been greatly exhausted ego depletion is experienced. At that point, a conservation process springs into action and the self abstains from further exerting itself (Hagger et al., 2010). Baumeister et al. (1999), expanded the theory to include depletion of resources that stem from deployment of coping efforts. They argued that stressful and traumatic events require coping efforts, further depleting one’s personal resources, in particular, volitional resources such as active coping modes. Coping requires volitional regulation and/or control of the self, and these attempts act to further drain available resources, especially when the stressful situation is ambiguous, uncontrollable, or highly aversive. Replenishment of such personal and coping resources, however, can be gradually accomplished through a variety of processes, including sleep, rest, exercise, diet, social support/activities and positive emotions. When an extensive degree of ego depletion occurs, the individual may experience severe health and mental conditions. A rich body of empirical research have been amassed to lend credence to most, but not all, of the postulates of the ego depletion model. For example, ego depletion was found to decrease guilt feelings, and such a decrease also resulted in participants’ reduced willingness to help others (Xu et al., 2012). Ego depletion was also reported to be associated in an intricate, and at times inexplicable, manner with unethical behavior under different conditions of self-control resources (Yam et al., 2014). Another study reported that athletes’ start reaction times decelerated following a depleting task, but not under non-depleting conditions (Englert and Bertrams, 2014). Despite the mostly supportive data available, the model’s veracity is, by no means, fully established (Hagger et al., 2010; Larquin and Miyake, 2017; Friese et al., 2019). Indeed, in a recent Special Issue of Social Psychology (Ego Depletion and Self-Control: Conceptual and Empirical Advances), Inzlicht and Friese (2019) concluded that despite supportive data from more than 600 studies, the model’s viability is still suspect and that concepts, such as motivation (or boredom) and willingness to exert effort, are just as viable in explaining the findings reported in the literature.

In their energy-based theory, Ryan and Frederick (1997) and Deci and Ryan (2000) argued that the energy available to the self is a salient indicator of a person’s health and motivation. They termed that energy *vitality.* Vitality, therefore, is an indicator of physical/somatic and mental/PEs that comprise subjective feelings of vigor, positive affect, psychological wellness, calmness, and better physical performance. It is the bridge to higher resilience to life stressors and minimization of disease activities (Ryan and Deci, 2008). In contrast, they claimed, negatively experienced levels of energy, or activation, reflect merely arousal states and include anxiety and anger, and are unrelated to subjective vitality. In support of their theory, Ryan and Deci developed their *Self-Determination Theory* (SDT) in which they argued that energy and vitality are enhanced when the essential psychological needs of autonomy (feeling volitional rather than controlled), competence (feeling effective) and relatedness (feeling connected and significant) are satisfied. Furthermore, they posited that autonomous self-regulation (volitional, or internally determined) expands one’s positive energy, vitality and productivity, and results in no ego depletion, while self-controlling efforts (non-volitional or externally determined) drain these energies, resulting in gradual ego depletion. Autonomous actions reflect one’s authentic values and interests. They are harmonious and efficient and demand less psychic conflict and inhibition and, therefore, are associated with physical and psychological well-being. Empirical support, from meta-analytic studies and systematic reviews, for SDT’s constructs (e.g., autonomous motivation, need support) has been obtained in various domains, including health promotion, disease management, and physical exercise (Teixeira et al., 2012; Ntoumanis et al., 2021).

An extension of Ryan and Deci’s (2008) vitality construct can be found in the model developed by Greenglass (2005, 2006). In her model, Greenglass suggested that vitality may be expressed in three separate, yet inter-correlated, venues namely, positive affect, vigor and “moving forward with life.” Positive affect is regarded as free-floating moods. Vigor, as traditionally viewed, is best exemplified by high degrees of future-oriented energy, activity, alertness, stamina, motivation and persistence. Finally, the life-associated forward movement is a reflection of one’s ability to consistently possess feelings and motivation about engaging in pleasure-generating activities and interpersonal relationships. Greenglass has provided preliminary empirical evidence, demonstrating the intimate relationships among the three components of vitality and conceptually similar constructs such as availability and use of social support, positive affect, proactive coping (i.e., coping efforts reflecting a belief that one has the potential and responsibility for improving oneself and the environment), and independent functioning, among various clinical and non-clinical populations, including patients in both hospitals and physical rehabilitation settings (Greenglass, 2006).

In his *Conservation of resources* (COR) theory, Hobfoll (1989; Hobfoll et al., 1996) stipulated that people seek to obtain, retain and protect resources which they regard as valuable. In a similar fashion to Lazarus’ theorizing about psychological stress, Hobfoll surmised that stress ensues when these resources are threatened, lost, or their investment fails to achieve adequate gain of future resources. Loss is viewed as the primary mechanism that launches stress reactions. Four categories of resources were suggested, object resources, condition resources, personal resources, and energy resources (e.g., time, money, credit, knowledge, information), the latter is conceived as facilitating the attainment of other resources. In this capacity, energy resources precede and motivate the success of the other resources. Following an encounter with a stressful event, the individual faces an increasingly depleted resource pool to combat stress and, thus, will have to resort to less active coping modalities and, instead, rely on more passive and emotion-focused coping efforts. When resource loss is severe and broad (depletion of energy is extreme), it may threaten the individual’s psychosocial functioning and physical survival (Hobfoll et al., 1996). In their efforts to empirically test the validity of the COR theory, Hobfoll et al. (1992) created a COR-Evaluation instrument to measure people’s available resources. The instrument purports to address resources used during experienced losses and gains. Although most of these resources sample financial, personal, and social domains, they also suggest the availability of energy-linked resources (adequate sleep, free time, stamina, motivation, etc.) that the theory postulates. Empirical support for the COR theory’s main constructs, including the association between the availability of work, social and personal resources and clinical outcomes, such as job burnout, PTSD, and CID-generated stress has been reported (Xanthopoulou et al., 2009; Hollifield et al., 2016; Roessler et al., 2019). Concerns about the theory construct veracity have also been raised (Halbesleben, 2006; Halbesleben et al., 2014). Data on the theory’s postulated energy resources are also scarce, and further refinement of its conceptual underpinnings and measurement procedures is needed.

Finally, a brief mention should be made of the contributions to stress, coping and PE, by Richard Lazarus. Although more implied that explicit in the theory, his reflections on PE, nevertheless, help illuminate our understanding of human emotions, cognitions, behaviors and coping strategies. For example, Lazarus (1966, 1993) theories of emotion and stress have allocated a significant role to PE in motivating human emotions and actions. In his earlier theorizing, Lazarus’ views on energy and adaptability were influenced by Duffy (1941) who regarded adaptational responses as driven by energy mobilization and having a distinct direction. Activation, rather than emotion, was seen by her as the prime mover of human functioning, and all behaviors were viewed as inherently motivated by the mobilized energy. Lazarus, however, abandoned this physics-flavored interpretation of energy, activation and behavior, and preferred a more psychologically informed (i.e., emotion-based, coping-related) perspective. In the context of coping with stress, he suggested that “health and energy are among the most pervasive resources in that they are relevant to coping in many, if not all, stressful encounters” (Lazarus and Folkman, 1984, p. 159). Indeed, an empirical support to the notion that energy level is linked positively to overall health status, and negatively to somatic symptoms and life hassles, was furnished by DeLongis et al. (1982). Lazarus (1993) further argued that people with CID possess less energy to expend on coping activities and, in particular, under stressful encounters that require extreme mobilization of energy. Finally, his tripartite appraisal model of stress (harm/loss, challenge and threat), intimates both the temporal direction and valence of energy. For example, harm/loss is associated with damage already done (energy is past-oriented), threat is related to anticipation of adversity (energy is future-oriented, reflecting personal concerns), while challenge is linked to effective mobilization and deployment of coping resources (energy is mostly present-oriented, reflecting anticipation of positively charged performance; Lazarus, 1993). The valence of the energy-fueling emotion depends on whether the potentially stress-producing context is judged to be favorable to goal attainment (yielding positive energies/emotions) or unfavorable to it (resulting in negative energies/emotions).

### Psychological Energy and Positive Psychology

The reach of PE extends to many of the constructs (e.g., personality attributes, coping resources) advocated and examined by proponents of the field of positive psychology. These include, among others: (a) resilience, reflecting an action-oriented approach that indicates one’s capacity to cope with adversity, adaptability to unexpected life changes, and rebounding from the aftermath of traumas and CID (Connor and Davidson, 2003); (b) sense of coherence, a tripartite construct referring to “a global orientation that life events one faces are comprehensible, that one has the resources to cope with the demands of these events, and that these demands are meaningful and worthy of engagement” (Antonovsky, 1987, p. 19); (c) self-actualization, a construct proposed and revisited numerous times over the past century, addressing the notion that individuals are motivated (have the need) to pursue and fulfill their unique potential in life (Maslow, 1968); (d) self-efficacy, regarded as a personal evaluation of one’s capacity to implement actions necessary for reaching goal-related outcome expectancies (Bandura, 1986); (e) hardiness, viewed as a tripartite personality trait (commitment, control and challenge), which permits the person to successfully approach stressful life events (Kobasa, 1979); (f) optimism, indicating a generalized outcome expectancy, or the perception that one is capable, through self-motivated behaviors, to move toward desirable goals (Scheier and Carver, 1985); (g) hope, referring to perceived motivation-driven capacity (agency) to use one’s pathways to achieve desired goals (Snyder et al., 2002); (h) posttraumatic growth, describing positive outcomes that emerge from the aftermath of trauma and loss, and includes experienced growth in personal strengths, relationships to others, appreciation of life, spiritual change, and perceiving new possibilities (Tedeschi and Calhoun, 1995); (i) flourishing (and thriving), encompassing an amalgam of personal strengths, including, finding purpose and meaning in life, relating to and engaging with other people, having positive emotions, and finding fulfillment in life accomplishments (Seligman, 2011); and (j) self-determination, composed of autonomy or self-control, competence or mastery, and relatedness or attachment to others, the three needs that make up Ryan and Frederick’s (1997) and Deci and Ryan’s (2000) SDT, which claims that people are intrinsically motivated to grow and change by achieving these universal psychological needs. Common to all these positive psychology-linked constructs is the belief that successful functioning emanates from: (a) possessing the capacity and resources to cope with adversity, crisis, trauma, and stressful life events; (b) being self-motivated to actively pursue and implement life goals; (c) having the motivation to grow in personal and interpersonal life domains, (d) finding purpose, meaning and benefits following adversity; and (e) exhibiting competence, mastery, autonomy, and commitment.

## Psychological Energy in the Context of Adaptation to Stress, Trauma and Chronic Illness and Disability

In this section an overview of the contributions of PE to models, directly relevant to adaptation to traumatic events, such as CID onset, is undertaken.

### Somatopsychological Perspectives and Psychological Energy

The concept of energy, or in its more focused version of vectors and forces, was first approached by the Lewinian theory-influenced somatopsychologists Barker et al. (1953/1977), in their classic volume *Adjustment to Physical Handicap and Illness*. They postulated that two sets of vectors operate on a person who experiences acute physical illness symptoms. One set of vectors (forces) stems from experienced symptoms that signify being ill and facing an uncertain diagnostic and therapeutic situation (endowed with a negative valence). The other set of vectors (forces) emanate from the wish to maintain renewed health and return to normalcy (endowed with a positive valence). This conflict is further exacerbated by the concurrently experienced overlapping situation where one set of vectors induces movement away from seeking diagnosis and treatment (a repelling vector, derived from fear of the unknown or seriousness of condition, and fear of therapeutic procedures), while the opposing set induces movement toward seeking diagnosis and treatment (an attracting vector, derived from the wish to avoid pain and discomfort, and seeking renewed health).^4^ The psychological tension generated by the interplay of these two sets of vectors is a product of the quality (positive or negative), direction, and intensity (valence) of these opposing forces, and ultimately determines if the individual seeks medical treatment (Shontz, 1975).

In her work, Wright (1983) took a somewhat different path to the narrative of adjustment to CID. The conflict between opposing vectors, assumed by Barker et al. (1953/1977), was diverted to the opposing forces of coping vs. succumbing to the impact of CID. More specifically, coping emphasizes what the person can do, and does, thus reflecting an active role (energy-infused) in molding one’s life. Succumbing, in contrast, emphasizes what the person cannot do, thus indicating the presence of a passive, or a victim, role (energy-depleted) that shapes one’s life. PE, as depicted by coping, is, therefore, equated with participation in valued personal and social activities, maintaining a hopeful perspective, and actively taking charge of one’s life (B. A. Wright, 1983). Similar conceptualizations can also be found in the work of Greer and Watson (Greer and Watson, 1987; Watson and Homewood, 2008), on adaptation to cancer (i.e., fighting spirit, signifying active acceptance vs. resignation, or fatalism, reflecting passive acceptance).

### Coping With and Reacting to the Onset of Chronic Illness and Disability

In his previously discussed approach to analyzing appraisal and coping stress, Lazarus (1966, 1993; Lazarus and Folkman, 1984) speculated about the nature of actions directed at such events, including those associated with physical traumas and psychological crises. He viewed direct actions as reflecting behaviors that are either aimed to attack the threatening agent or avoid it. These actions were seen as ontologically primitive forms of coping modes. An attacking mode (active confrontation of the threating agent) is fueled by the emotion of anger and is outwardly directed. In the context of reacting to CID, it coincides with blaming others for CID onset or lack of responsiveness to treatment (Shontz, 1975). In contrast, an avoiding mode (retreating from a threatening agent) is triggered by the emotions of fear and anxiety that are inwardly directed. In the context of experiencing CID, it coincides with blaming oneself for CID onset or failure to medically improve, and feeling guilty about the failure (Shontz). Aggressive actions promote further growth of goal-oriented behaviors, and the *energy* released by these vigorous activities is put to productive use that serves to minimize functional limitations and acquire additional skills (Shontz, 1975). Avoidance, in contrast, lacks the necessary energy to produce useful growth and often results in stagnation and denial-like maneuvers. Lazarus and Shontz imbue psychological reactions triggered by traumatic events, such as the onset of CID, with the recognizable indicators of PE direction (its vector; inwardly or outwardly directed), magnitude or valence (its force or strength) and quality or attribute (positive or negative).

In a similar vein, Livneh (1986; Livneh and Siller, 2015) argued that psychological reactions to the onset of CID could be envisioned as possessing energy, direction and impetus. Several often-cited reactions were examined, including anxiety, denial, depression, internalized and externalized aggression/anger, and acceptance. Anxiety, triggered by the onset of CID, is associated with diffuse and potent levels of energy (cognitive flooding and disorganization) in which energy is directed inwardly to protect (often unsuccessfully) the self. In denial, the energy available to the self is used to suppress or negate the ramifications, and even the presence, of CID reminders (Breznitz, 1983; Livneh, 2009). Energy is selectively deployed to minimize and avoid unacceptable realities. Accordingly, energy direction alternates, and shifts between blocking unwelcome messages from the external world and seeking to subterfuge the psyche’s recognition of these painful realities.

When a person experiences depression, as well as when anger is directed inwardly, energy levels plumet and the individual socially withdraws, loses interest in previously engaged activities, experiences long periods of fatigue, and resigns to his or her new situation. Feelings of self-blame, shame and guilt arise and indicate externally blocked PE (Shontz, 1975; Pines, 1990). The reduced energy is now directed inwardly, toward the injured psyche, and is negatively saturated. An increase in energy level and reversal of direction become evident, however, when the person’s response to CID reflects externally oriented anger. Such a reaction is best exemplified by direct and active anger expression, including blaming others for CID onset; riling against slow progression to treatment; and retaliating against outside agents (Shontz, 1975; Livneh, 1986). Energy, then, is outwardly directed and is negatively imbued.

Acceptance of CID (i.e., actively acknowledging its present and future implications, yet not being deterred by its functional restrictions and possible impact on daily activities), is best conceived as indicating cognitive and emotional reorganization, renewed and balanced orientation toward both self and the world, and reconciliation between inner needs and wishes, and environmental demands. Energy direction now reflects a balanced investment, of positively valenced-energy, between internally oriented (e.g., physical, psychological) and externally oriented (e.g., social, vocational) processes (Livneh, 1986).

### The Energy Psychology Model and Adaptation to Chronic Illness and Disability

Finally, concepts borrowed from the emerging field of energy psychology (EP) further highlight the relationships between the presence of energy fields, including electromagnetic signals and brain waves, and adaptation to CID. Although discussion of the field of EP is beyond the scope of the present paper, EP does suggest that intrapersonal disturbances, including medical and bodily conditions (e.g., limb amputation) and psychiatric disorders (e.g., PTSD, obsessive-compulsive disorder, mood disorders), and trauma-linked responses (e.g., anxiety, anger, grief, shame, guilt, rejection) are related to disturbances in the adjacent environments (interpersonal and extrapersonal), including the body’s biochemical and electromagnetic energies and its energy fields (Gallo, 2004; Feinstein, 2012, 2021). As such, EP is conceived as a set of physical, cognitive, behavioral, psychodynamic and therapeutic modalities (e.g., imaginal exposure therapy, acupuncture, mindfulness) designated to target emotions, cognitions and behaviors associated with, among others, CID-generated psychosocial reactions. More specifically, EP employs a set of structured mechanisms, utilizing tapping on specific acupoints, during imaginal exposure, to produce electrical signals and subsequently reduce brain arousal (Feinstein, 2012). EP’s two most commonly employed experimental techniques, namely, Emotional Freedom Techniques and Thought Field Therapy, apply stimulation of (i.e., tapping on) sensitive skin regions to regulate stress and achieve personal transformation. Both are rooted in Eastern philosophy and the practice of acupuncture (Stapleton, 2014). EP further maintains that a bidirectional causal link exists between the human body and mind, and energy fields (Leskowitz, 2014, 2018). Biopsychosocial energy imbalance, or blockage, often follows physical and/or psychic trauma, and influences both body and mind and is directly responsible for the occurrence of both somatic (e.g., pain, discomfort) and psychological (e.g., PTSD, anxiety) symptoms. Phantom limb pain, often associated with traumatic limb amputation, is regarded by EP proponents as a typical example of blocked energy responsible for the appearance of both somatic pain and its psychic counterpart (PTSD). This type of pain is conceptualized as an energy sequela encompassing both physical and emotional traumas (Leskowitz, 2014). A growing body of clinical and empirical literatures has now been accumulated, integrating time honored Eastern healing practices and contemporary scientific knowledge, to support the viability of EP as a useful, even if in need of further refinements, concept.

In sum, then, and adopting a broader and more philosophically spawned perspective, the existence of pre-CID psychosocial homeostasis, derived from years of life-informed fine-tuning must, following the shattering experience of personal trauma and CID onset, be abruptly reconfigured and extend to a larger and previously seldom tested life domains. Thus, earlier effective management of these life domains confronts both dilution (spreading over a larger number of domains) and uncertainty (some domains were previously underutilized). These newly experienced (and previously only minimally energy-importing) domains now surface, and must be confronted, because of the need to reduce CID’s deleterious impact on one’s life. These domains include, among others, health maintenance, pain management, medication administration, mobility training, and financial burden. Adopting an analogy, borrowed from the field of physics, it can be argued that following the onset of a sudden CID, the geometry of one’s lifespace has been warped. This sense of psychological warping of the spatial structure (the environment within which the person functions) and of the perception of time flow, is an attempt to accommodate the myriad newly impinging physical, perceptual, proprioceptive, nociceptive, social and psychological forces that the individual must now cope with, and for which the reservoir of available energy may no longer be sufficient.

## The Measurement of Psychological Energy

The measurement of a construct as abstract and complex as PE presents researchers with a formidable task whose successful dismantling requires much creativity and insight. Researchers appear to have been equal to the task. In this section, a review of the most commonly adopted measures of PE is attempted. A wide range of energy-measuring tools have been proposed. They can be conveniently grouped into the following five broad categories: (a) physiological indices, such as cardiovascular and glucose levels functioning; (b) subjective measures of PE that typically rely on perceived vitality, vigor, or their counterparts, namely, fatigue, exhaustion, burnout, and ego or energy depletion; (c) proxy measures derived from clinical/psychiatric indicators, such as depression, anxiety, and anger; (d) energy-linked measures, derived from more enduring personality characteristics, such as resilience, hardiness, locus of control, and self-efficacy; and (e) coping modes (with stress, adversity and CID) which, when viewed in the context of PE, suggest a conceptual resemblance to the operation of adaptive, effective, active and flexible coping skills and strategies, as well as between lower levels of PE and non-adaptive, ineffective, passive and inflexible coping strategies.

### Physiological Measures

Two primary measures have been suggested, namely, cardiovascular reactivity and blood glucose level. Other physiological indices of energy expenditure, less often employed, include galvanic skin response, respiration, and skin temperature (Altini et al., 2014). Cardiovascular reactivity refers to the responsiveness of such indicators as heart rate, systolic (and, less frequently, diastolic) blood pressure, and vasoconstriction, among others, to externally manipulated stimuli (Cacioppo and Petty, 1982; Wright, 1987; Huang et al., 2020). These measures serve as indicators of the person’s physiological state of readiness for action and energy mobilization. Support for this assumption has been garnered from animal studies, and research on human infants and adults (Wright, 1996; Gendolla et al., 2012). Research on blood glucose levels has, similarly, supported the notion that efforts at self-regulation, such as managing conflicts between equally important motivations, results in drop of blood glucose levels (indicating reduced energy), and these reductions predict poorer behavioral performance in subsequent acts of self-regulation (Gailliot et al., 2007; DeWall et al., 2008). These researchers also demonstrated that when blood glucose level was restored (by sugary drinks), ego depletion levels (measured by behavioral indicators, such as willingness to help others, volunteering, etc.) were effectively neutralized. Further research, however, is needed to confirm the stipulated concepts underlying this theory.

### Proxy Psychiatric/Clinical Measures

Items derived from traditional psychiatric and psychological measures can be, and have been, used as proxy indicators of energy. Well beyond the scope of this paper to describe thoroughly, a brief mention of a few of these indicators is warranted. Examining the affective domain, Ryan et al. (1995), argued that subjective vitality (as a PE equivalent) should be negatively associated with certain indicators of depression, including anergia and amotivation, as well as perceived external locus of control. Penninx et al. (2000), in their discussion of emotional vitality argued that it is a product of both positive affect (i.e., mastery, happiness), as well as low negative affect (i.e., depression, anxiety). Many of the measured indicators of depression (e.g., loss of appetite, poor sleeping, getting tired easily, feeling powerless, having difficulty concentrating, experiencing decreased motor activity) and selected indicators of anxiety (e.g., dwelling on past mistakes, avoiding new or unfamiliar situations, avoiding challenges, withdrawing from social gatherings) could serve as proxies for low energy. In a similar vein, indicators of anger, or aggressive behavior, are traditionally regarded as conveying higher, albeit diffuse, arousal and energy levels (Siegel, 1986; Spielberger and Reheiser, 2010). Representative proxy indicators of elevated PE-linked anger could be observed in: (a) verbal manifestations (being argumentative, criticizing others, insulting or yelling at others); (b) physical manifestations (physically hitting others, breaking items, acting out frustrations); and (c) cognitive-affective manifestations (seeking revenge, feeling furious, harboring grudges).

### Self-Report Measures of Energy-Like Constructs

Among this heterogenous group of measures the following are reviewed: specific vitality and vigor indices, fatigue and exhaustion markers, and finally ego- and energy-depletion instruments, that address vitality and fatigue in specific settings, including health and occupational ones (see Table 2).

**TABLE 2:** Primary measures of psychological energy.

Measure	Contributing authors	Pertinent (sub)scales	Representative items
Vitality scale	Ryan and Frederick	Vitality (state and trait versions; each 7 items)	“I feel alive and vital”; “I don’t feel very energetic”
Profile of Mood States (POMS); SV-POMS	McNair, Lorr, and Droppelman; Shacham	Vigor-Activity scale (8 items); Fatigue-Inertia scale (7 items)	VA = “active” “energetic” “vigorous”; FI = “worn out” “fatigued,” “exhausted”
Mental Adjustment to Cancer (MAC) Scale	Greer and Watson; Watson and Homewood	Fighting Spirit (16 items), Helplessness/Hopelessness (6 items) and Fatalism (8 items)	FS = “I won’t let cancer beat me, I’m trying everything to get better”; H/H = “There is nothing they can do, I’m finished”; F = “It’s cancer, I don’t dwell on it, try not to think about it”
The Short-Form-36 Health Survey Questionnaire (SF-36 HSQ)	Ware and Gandek	Vitality Scale (4 items)	“Did you feel full of pep?”; “Did you feel worn out?”
Modified Fatigue Impact Scale (MFIS)	Fisk et al.	3 fatigue subscales (physical, cognitive, psychosocial; 21 items)	“I have been less alert”; “I have needed to rest more often or for longer periods”
Fatigue Severity Scale (FSS)	Krupp et al.	Fatigue (or Energy Level) Scale (9 items)	“My motivation is lower when I am fatigued”; “Fatigue interferes with my work, family, or social life”
Multiscore Depression Inventory (MDI)	Berndt, Petzel, and Berndt	Fatigue subscale (12 items)	“It seems like I am always tired”; “My energy level is usually high”
PROMIS-Fatigue-SF	Cook, Molton, and Jensen	Fatigue Scale (10 items)	“I feel fatigued”; “I have to limit my social activity because I’m tired”
Visual Analogue Scale for Global Vigor and Global Affect (VAS GV and VAS GA)	Monk	1 scale, Visual Analogue Vigor Scale (4 items)	“How alert do you feel?”; “How sleepy do you feel?”
Lee Fatigue and Energy Scales (VAS-F)	Lee, Hicks, and Nino-Murcia	2 scales, 18-item, bipolarly anchored, Visual Analogue Scales; Fatigue (13 items) and Energy (5 items) subscales	F = “Not at all tired—extremely tired”; “Moving my body is no effort at all“—“moving my body is a tremendous chore”; E = “Not at all energetic—extremely energetic”; “Not at all active—extremely active”
Maastricht Questionnaire	Appels, Hoppener, and Mulder	1 Scale, Vital Exhaustion, trichotomically structured (21 items)	“Do you often feel tired?”; “Do you have the feeling that you can’t cope?”
Utrecht Work Engagement Scale	Schaufeli	3 work-related dimensions; 1 Vigor subscale (6 items)	“At my work, I feel that I am bursting with energy”; “At my job, I feel strong and vigorous”
Maslach Burnout Inventory	Maslach and Jackson	4-factor inventory; 1 Emotional Exhaustion subscale (9 items)	“I feel emotionally drained from my work”; “I feel used up at the end of the work day”

#### Vitality and Vigor

Subjective vitality has been defined by Ryan and Frederick (1997) as “one’s conscious experience of possessing energy and aliveness” (p. 530) and regarded it as an indicator of an organismic state of intrinsic motivation. They developed a 7-item subjective *vitality* scale (including both trait and state versions), whose psychometric soundness was supported by findings demonstrating its predicted association with measures of psychological well-being, self-actualization, self-determination, self-esteem, self-efficacy and body functioning (all positively), and psychological distress, depression, and anxiety (all negatively). It was also related to reports of having better body functioning and fewer physical symptoms. Decreased vitality was linked to higher levels of experienced pain, and was evident among chemotherapy-treated cancer patients (Ryan and Frederick, 1997; Ryan and Deci, 2008).

Three additional psychosocial instruments that include items depicting vitality and vigor include the Profile of Mood States (POMS); McNair et al. (1971), and its abbreviated form, the Shorter Version POMS (SV-POMS; Shacham, 1983), the Mental Adjustment to Cancer (MAC) Scale (Greer and Watson, 1987; Watson and Homewood, 2008), and the Short Form of the Health Survey Questionnaire (SF-36 HSQ; Ware and Gandek, 1998; World Health Organization, 2005). Among its 65-item, 6-mood/affective states, adjective rating scale, the POMS includes measures of vigor-activity and fatigue-inertia. Numerous empirical studies have supported the validity and reliability, as well as the diagnostic utility, of the POMS vigor and fatigue scales (e.g., O’Connor, 2004; Bourgeois et al., 2010). The MAC focuses more squarely on adaptation to cancer. It is a 40-item, 5-subscale, self-report measure. One of its subscales, namely, *fighting spirit* indicates the availability of positive energy to combat the impact of cancer. In contrast, two other subscales, namely, *helplessness/hopelessness* and *fatalism* indicate the presence of depleted energy to manage the aftermath of cancer diagnosis. Ample empirical support of the clinical utility and validity of the MAC has been aggregated over the past four decades (Watson and Homewood, 2008). Third, a health-related QOL-type scale, the SF-36 HSQ, provides relevant information on PE in the form of its Vitality scale (one of eight scales composing the SF-36 HSQ). Hundreds of studies have demonstrated the SF-36 HSQ’s, and its Vitality scale’s, viability and psychometric strengths, and consistently affirmed its capability to predict subjective well-being, and physical and social functioning among medical patients (O’Connor, 2004; Bjorner et al., 2007).^5^

Several researcher-improvised measures have also been reported. For example, in their investigation of the relationships among health status and major stressful life events, DeLongis et al. (1982) adopted a revised version of the Health Status Questionnaire, which includes a subscale of respondents’ perceived energy levels. Items focused mostly on physical energy indicators (e.g., sleep, fatigue, energy level). A composite score of “energy level” was created, and as hypothesized, was associated with health status (positively), as well as somatic symptoms and magnitude of daily hassles (negatively).

Finally, two visual analog techniques were applied to energy measurement. In the first, Monk (1989), proposed a visual analog scale to measure both global vigor and global affect. The measures yield separate summary scores for global vigor or global affect. Preliminary data on the measure’s reliability and validity were provided by the author. A second visual analog measure [the Lee Fatigue and Energy Scales (VAS-F)] consists of bipolarly anchored descriptors that purport to assess both fatigue and energy (Lee et al., 1991). Support for the psychometric soundness of the VAS-F has been obtained among a wide range of medical samples, including people with sleep disorders, cancer, chronic illnesses and HIV/AIDS (Lerdal et al., 2013).

#### Fatigue and Exhaustion

Specific fatigue-assessing measures are also relevant here. They include, among others, the 3-subscale (physical, cognitive, and psychosocial) Modified Fatigue Impact Scale (MFIS; Fisk et al., 1994) and the unidimensional Fatigue Severity Scale (FSS; Krupp et al., 1989). Fatigue measuring subscales, embedded in larger instruments also exist and include the 118-item, 10-subscale, Multiscore Depression Inventory, hosting a Fatigue subscale (MDI; Berndt et al., 1980), and the PROMIS Fatigue Short Form (PROMIS-F-SF; Cook et al., 2011). Empirical findings, obtained from various CID populations, have demonstrated the validity and usefulness of these measures, including the hypothesized association to depression and maladaptive beliefs (positive), resilience and perceived physical health (negative; Cook et al., 2011; Terrill et al., 2016; Bassi et al., 2019).

A promising measure of energy depletion is the Maastricht Questionnaire, developed to assess feelings of vital exhaustion (Appels et al., 1987), among Dutch survivors of myocardial infarction. Findings showed that feelings of vital exhaustion were significantly higher among MI survivors than in a control group. Additional support for the validity of the Vital Exhaustion construct was reported among burn-injured people (Fauerbach and Perry-Parish, 2018) who, following stressful therapeutic sessions, experienced reduced energy and increased levels of fatigue and helplessness.

### Context-Specific Energy Measures

Finally, two context-specific, energy-measuring scales, focus on occupational settings. A Dutch measure, the Utrecht Work Engagement Scale, purports to provide scores on three work-related dimensions, including vigor, dedication, and absorption, and serve as a global indicator of occupational well-being (Schaufeli et al., 2002). The scale is a 3-subscale, 17-item measure, one of which is a Vigor subscale. The scale’s 3-factorial structure and temporal stability have been consistently supported (Mills et al., 2012). The second measure, explores work burnout, and was developed by Maslach and Jackson (1981; the Maslach Burnout Inventory). One of the inventory’s four factors is titled “Emotional exhaustion,” and is composed of items whose tone indicates feelings of being emotionally exhausted by one’s work. The authors provide substantial support for the measure’s reliability and validity.

### Personality- and Coping-Driven Energy-Equivalent Measures

Infrequently explored in the health and rehabilitation literature, energy-proxy indicators that reflect stable personality traits and characteristics are, nevertheless, potentially useful measures to consider. Although numerous examples of energy-proxy, or indirect, indicators (i.e., items and scales) from the literature can be provided, three notable ones (resilience, hardiness and locus of control) should be considered. First, resilience has been measured with the Connor-Davidson Resilience Scale (CD-RISC; Connor and Davidson, 2003). Among its 5-subscale, 25 item, self-report scale, the following items suggest energy content: “Can deal with whatever comes,” and “best effort no matter what.” Similarly, the Hardiness Scale (Bartone et al., 1989), a 45-item, self-report scale, was developed to tap the three components of hardiness, namely, commitment, control, and challenge. Items reflective of energy-like qualities include “by working hard you can always achieve your goals,” and “I often wake up eager to take up my life wherever it left off.” Finally, the Internal-External Locus of Control Scale (IE-LOCS; Rotter, 1966), is a widely used unidimensional, 2-item paired, measure of generalized expectancies regarding the causation of outcomes. Examples of items indicating internal (i.e., the person’s own actions and attributes determine the experiences depicted in the item), include “becoming a success is a matter of hard work,” and “when I make plans, I’m almost certain that I can make them work.”

An additional source of energy-implicating measures can be found in the vast literature on coping with adversity, trauma, stress, and CID onset. Indicators of both adaptive (or effective), as well as non-adaptive (or non-effective) coping strategies may be regarded as reflective of availability, or lack thereof, of PE to manage stressful events (Lazarus and Folkman, 1984; Rozanski and Kubzansky, 2005; Martz and Livneh, 2007). For example, Rozanski and Kubzansky (2005) argued that a sense of vitality, which they viewed as a composite of positive energy and positive emotions, is directly linked to effective coping skills (manifesting coping flexibility) and emotional well-being (manifested by psychological competence and emotional flexibility). They further maintained that various physical (e.g., medical impairment) and psychological (e.g., stress, poor coping skills, negative emotions) factors act to de-energize the individual. Positive emotions and effective coping skills, in contrast, provide positive energy. The ability to flexibly adopt a wide range of adaptive coping strategies (e.g., problem-solving, emotional regulation, social support seeking), and fine-tune them in accordance with the controllability, predictability and manageability of the situation serves to both increase one’s positive energy, as well as maximize the prospects of achieving emotional well-being and improved QOL (Martz and Livneh, 2007). It stands to reason, then, that scale items reflecting adaptive and flexible coping strategies (e.g., ‘‘thought of different ways to deal with the problem,’’ ‘‘made a plan of action and followed it’’) should be associated with higher levels of PE, while those indicating non-adaptive and inflexible strategies (e.g., ‘‘kept away from people,’’ ‘‘tried to deny how serious the problem really is’’) would be linked to low and negative energy.^6^ Further research, however, is needed to provide incontrovertible evidence in support of these speculations.

## Physical and Psychological Energy: Further Thoughts

The manifestation of physics-informed energy, in its several forms, within the field of psychology in general, and psychosocial adaptation to trauma, crisis, and CID onset more specifically, should not come as a surprise to theoreticians and researchers. Although speculative in nature, the broad similarities between energy, as observed in physics, and a number of its psychological counterparts, will be briefly explored in this concluding section.

### Parallels Between Physical Energy Forms and Psychological Constructs

Among the many forms of energy described by physicists, potential, gravitational and kinetic energy are the most commonly encountered. By potential energy physicists refer to energy available for doing work. It is, therefore, energy stored in objects, and as objects fall (gravitationally induced) their potential energy is converted into kinetic energy, that is motion (Krauss, 2012). In psychology, the equivalent of potential energy refers to energy (e.g., vitality, vigor, motivation) stored in positively valenced personality traits and resources that serve to combat stress, trauma and adversity. They include constructs such as hope, optimism, self-esteem, self-efficacy, resilience, sense of coherence, mastery, and hardiness (see earlier discussion). These relatively stable, positively valenced characteristics are implicated in providing the engine necessary to fuel life functioning. A second form of energy, gravitational energy, is a derivative of potential energy, and refers to the mutual energy exerted by two (or more) massive objects on each other. It is the potential energy stored in a gravitational field in which these objects are located. When an object falls down in a gravitational field, energy is released. Gravitational energy, therefore, is always *negative* in nature (Krauss, 2012). When viewed from the psychological perspective of confronting trauma and crisis, gravitational energy takes the form of the energy released by the impact of the traumatic experiences (e.g., CID; negatively valenced). The psychological (gravitational and negative) energy generated by the impact can be equated with what models, such as illness intrusiveness (Devins et al., 1983; Devins, 2010), disability centrality (Bishop, 2005), illness centrality (Charmaz, 1983), illness self-concept injury (Morea et al., 2008), survivor centrality (Helgeson, 2011), injury centrality (Shiloh et al., 2014), disability self-concept (Bogart, 2014), and (negative) illness self-representations (Leventhal et al., 1997) view as the perceived psychological magnitude of CID. Indeed, the psychological impact experienced by an individual, following the onset of CID, can be regarded as a set of disruptions of, and upheavals (e.g., negatively valenced energy) affecting, one’s self-concept, subjective well-being, life style, daily activities, functional level, and anticipated therapeutic outcomes. A final energy-linked form is that of kinetic energy. Its primary manifestation is motion. It indicates an energy transferred to an object, and thus setting the object in motion (Krauss, 2012). The equivalent of such energy transfer from a source to an object, in the field of psychology, can be found in energy infused into launching of appraisal modes and coping strategies to minimize the impact of trauma, crisis, and adversity. The valence of the released energy determines the outcomes accomplished. Positively valenced energy is associated with the deployment of adaptive or effective coping strategies (e.g., planning, problem-solving, seeking social support), while negatively valenced energy is linked to launching non-adaptive or non-effective coping strategies (e.g., avoidance, behavioral disengagement, wishful thinking; Martz and Livneh, 2007).

### The Dimensions of Psychological Energy

Structurally, PE is comprised of at least three quasi-independent dimensions. These include: direction, quality (or nature), and magnitude.

#### Direction

Psychological energy can be directed inwardly or outwardly. As discussed earlier, internally oriented PE indicates energy whose vector points toward an object (the individual). In contrast, externally oriented PE is manifested as a vector pointing toward the environment (away from the individual). Examples of inwardly directed, coping-linked energy, include self-controlling, positive reappraisal, self-blaming, and wishful thinking; and among CID-triggered psychological reactions, anxiety, depression, and shame, indicate internally oriented PE. In contrast, outwardly directed coping energy efforts include confronting, seeking social support, other-blaming, and direct action, while CID-triggered responses of anger and aggressive behavior, also reflect externally oriented PE.

#### Quality

Psychological energy can also be depicted by its inherent quality-defining characteristics. In physics, quality is, at best, vaguely understood, and typically refers to the type or form of energy described (e.g., potential, kinetic). In psychology, the term refers to a degree of attractiveness, pleasantness or positiveness of an event, experience, or activity. Efforts associated with positive PE refer to those directed at managing or defusing the impact and implications of a traumatic experience, including CID. In contrast, efforts linked to negative PE are those seeking to avoid, deny, or subterfuge the authenticity and impact of traumatic events. Examples of positively saturated coping strategies include planful problem-solving, emotional control, seeking social support, and cognitive restructuring, while acceptance and meaning finding, indicate positively toned psychological reactions. Examples of negatively saturated coping strategies, include wishful thinking, avoidance, social withdrawal, and substance use, while anxiety and anger represent negatively toned psychological responses.

#### Magnitude

Energy, and PE, can also be defined by their strength or valence. Unlike the first two qualities which are essentially dichotomous in nature (internal vs. external orientation, positivity vs. negativity in character), magnitude obeys a continuous scale of values, ranging from low or weak to high or extreme. Energy levels on this scale, range, for denial, from partial or mild denial to extreme or complete denial (Breznitz, 1983; Kortte and Wegener, 2004), for acceptance (periodically referred to as acknowledgment), from passive to active acceptance (Shontz, 1975; Livneh, 2021), and for non-adaptive coping, such as substance abuse, from minor (e.g., occasional social alcohol drinking; use of recreational drugs) to major (e.g., daily alcohol drinking; use of hard drugs). More complex, multilayered constructs such as illness intrusiveness, disability centrality, and illness self-representations, could be assessed through their impact on one’s LS and daily functioning. Accordingly, they may range from minor or partial (e.g., illness intrusiveness’ impact on one’s LS is mostly successfully contained) to major or extensive (e.g., illness intrusiveness affects most life domains and functional areas, and is practically uncontained).

### Conservation of Energy

Underlying the law of conservation of energy, in physics, is the principle that energy (in an isolated system) cannot be destroyed or created, over time. However, it can be altered among various forms of energy, for example, between potential and kinetic energies, or thermal and mechanical energies (Krauss, 2012). In biology, or more specifically the human body, conservation of energy takes a somewhat different form, since living organisms are not isolated systems but are interactive with their external environments (Davies, 2019). Four forms of energy fuel the functioning of most living organisms, and maintain a quasi-stable energy level. These include: (a) thermal (body temperature regulation), (b) mechanical (ambulation, position alterations), (c) chemical (molecular and cellular functioning), and (d) electrical (neural conductivity and cerebral activity; Davies, 2019). This principle, however, loses its effect when applied to PE. Life events such as stresses, crises, and traumas are often unpredictable, follow widely different trajectories, and require a substantial and often fluctuating deployment of input and output of PE. In the context of an open system, in which life exists and consciousness prevails, an orthodoxly applied principle of energy conservation is superfluous. However, since the pioneering reflections of Lewin (1935), the literature has strongly suggested that PE obeys the rules of energy conservation and being of limited capacity. The theories, discussed earlier, of Selye (1975; the general adaptation syndrome), Hobfoll (1989; COR, including energy), Brehm et al. (1983; energy mobilization), Baumeister (2002, 2003; self-regulation/control), and Ryan and Deci (2008; self-determination), all posit the existence of boundaries on the expenditure and replenishment of PE (at times, referred to as vitality, vigor, etc.). More specifically, all theories advocate the following: (a) individuals (and organisms, in general) possess a limited, finite and depletable amount of energy (resources, reservoir); (b) stressful events tax the energy reservoir and eventually deplete it, and the onset of CID broadens the range of experienced stresses and losses, thus further reducing the energy reservoir; (c) continuously mobilized coping efforts, triggered by stressful and traumatic events, further deplete energy resources; (d) when energy resources are threatened, or are substantially reduced, stress ensues, thus creating a vicious cycle, requiring more active (problem-focused) coping efforts to manage the stress, which in turn further deplete the energy reservoir; (e) self-controlling, psychologically restrictive, activities serve to exhaust and deplete energy resources; (f) personal needs, wishes, values and goals fuel PE, and its psychological counterparts, arousal and motivation; and (g) PE, comprised of subjective feelings of vitality, vigor, and being active, is linked to emotional well-being and perceived life-satisfaction.

### Denial and Psychological Energy

An additional clinical manifestation, often reported among survivors of traumatic experiences and sudden onset CIDs, is that of denial. In contrast to the energy-depleted states of clinical depression or anxiety, the adoption (mostly unconscious) of denial suggests an alternating inwardly and outwardly directed energy, comprised of suppression and negation of one’s CID, its extent, severity and future implications (Breznitz, 1983; Horowitz, 1983; Livneh, 1986, 2009). Denial-bound energy processing is a complex, multifactorial, and posing an almost insurmountable measurement challenge. It can be speculated that denial results in a certain amount of energy deployed to protect the self from the threatening realization of a life-altering loss or traumatic event. Psychodynamically oriented models (e.g., Shanan, 1989), likewise, suggest that denial is a necessary mechanism toward internal redeployment of psychic energy, and its reinvestment in new personal and interpersonal pursuits, thus leading to future energy-efficient engagement, and eventually adaptation. Such energy possesses both quantitative (magnitude, direction) and qualitative (nature, attribute) properties. Further research, however, is required to better understand the relationships between the often empiricism-defying nature of denial and PE.

### Reviewing Adaptation to Chronic Illness and Disability Constructs Through the Prism of Psychological Energy

Several of the constructs employed by health, rehabilitation and crisis and trauma psychologists can be, conveniently, decomposed into the three primary dimensions of PE (i.e., direction, quality and magnitude).^7^ Such a dimensional decomposition may have both clinical and research merits.

For example, constructs linked to adaptation to CID can be broadly classified under the following domains: (a) appraisal (i.e., pre-coping modalities, such as harm/loss, threat and challenge); (b) meta- and specific- coping strategies (e.g., engagement and disengagement coping as representative of broader or higher order strategies; planning and avoidance as reflective of specific or first order strategies); (c) quasi-coping modes (e.g., benefit finding, meaning making, posttraumatic growth); (d) perceptions of CID impact (e.g., illness intrusiveness, disability centrality, illness self-perceptions); (e) mostly enduring personality traits or attributes (e.g., resilience, neuroticism, optimism vs. pessimism, hope); and (f) “raw” psychological responses to trauma, crisis and loss (i.e., anxiety, depression, anger; Livneh, 2021). For example, a construct such as problem-focused coping can be decomposed into its three constituent energy elements as follows: (a) *direction*: externally oriented; (b*) quality*: positive/adaptive (in the majority of cases); (c) *magnitude*: medium to high. Similarly, a construct such as depression can be subclassified into the following elements: (a) *direction*: internally oriented; (b) *quality*: negative/non-adaptive; (c) *magnitude*: variable (depression can range from mild to severe, however, its magnitude can be specified in individual cases). Finally, a more intricate construct such as posttraumatic growth can be considered as composable into: (a) *direction*: both internally and externally oriented, as inferred from its functional components (e.g., spiritual change and relating to others, respectively); (b) *quality*: positive/adaptive (although pseudo-growth due to the operation of mechanisms such as wishful thinking and denial must also be considered); (c) *magnitude*: variable [as measured by the PTG Inventory (Tedeschi and Calhoun, 1996), and ranging from low to high].

## Summary

In this paper, the construct of PE was examined. The origins of this construct can be traced back to the seminal contributions of Sigmund Freud, Kurt Lewin, and Hans Selye, during the early and middle of the 20th century. They perceived PE as reflective of certain inherent psychic drives and forces that obey the principle of energy conservation. However, this functionally conserved energy, may undergo dynamic alterations, and in an open system such as human life, can be transformed into physical, behavioral and psychological (emotional, cognitive) energies, and, therefore, subject to temporary depletion, or exhaustion, and in need of replenishment, due to one’s finite and restricted reservoir of energy. Later contributors further expanded upon the nature and mobilization of PE, and equated it with such constructs as vigor, vitality, motivation, productivity, directed action, need satisfaction, and goal pursual. Energy depletion has also intimated depletion of personal/ego resources. Stressful and traumatic situations, as well as overextended use of coping efforts were also regarded as personal resource-depleting events. These resources, however, were believed to be replenishable, by physical, psychological and social means. The belief in the existence of PE resources and operations, was further buoyed by the emergence of the field of positive psychology. Constructs such as resilience, sense of coherence, self-efficacy, hardiness, and personal growth, although not necessarily and causally spawned by this field, nevertheless, are conceptually linked to the availability and positive contributions afforded by energy resources.

Psychological energy has been, likewise, intimately associated with dynamic frameworks of adaptation to CID. Earlier psychosomatically derived contributions, suggested that, following the onset of CID, the ensued psychosocial dynamics is best characterized by diametrically opposed forces, or vectors. These forces indicate the tension created by the tug-of-war between repelling forces (indicating negative valence, such as avoidance of diagnostic implications) and attracting forces (indicating positive valence, such as seeking health and returning to normalcy), following CID onset. A second, clinically informed, atheoretical, framework focuses on the direction and magnitude of post-CID psychological reactions, charted by these energy forces. These energy-driven, vector-like reactions are viewed as being either internally oriented (in depression, anxiety), or externally oriented (in anger). A more balanced energy distribution is also recognized (in acceptance, and some forms of denial).

Another area, in need of further conceptual and empirical refinement, centers around the numerous, but epistemologically frail, efforts to measure PE resources and dynamics. These efforts rely on a wide range of physiological, clinical, behavioral, perceptual and quasi-personality-linked PE indicators. Although showing much promise, these measures require a more streamlined, informant-supported, cross-disciplinary effort to examine their convergent validity and clinical utility. Finally, to better understand the complex relationships between physics-originated energy and PE, future research must explore: (a) the symmetries (or asymmetries) between the different forms of time-honored physical energy and their PE counterparts, including the temporal-spatial dimensions inherent in both physical and PEs (direction, quality, magnitude, duration); (b) the fuzzy, yet promising, construct of energy conservation, and its relationships to traumatic events and life-changing CIDs; and (c) any higher-order levels of analysis that seek to investigate, in more detail, the process of adaptation to traumatic experiences, including CID onset, and using PE as their primary driving force.

## Author Contributions

The author confirms being the sole contributor of this work and has approved it for publication.

## Conflict of Interest

The author declares that the research was conducted in the absence of any commercial or financial relationships that could be construed as a potential conflict of interest.

## Publisher’s Note

All claims expressed in this article are solely those of the authors and do not necessarily represent those of their affiliated organizations, or those of the publisher, the editors and the reviewers. Any product that may be evaluated in this article, or claim that may be made by its manufacturer, is not guaranteed or endorsed by the publisher.

## Footnotes

1 Various forms of energy exist and they include: potential (position or location energy), kinetic (energy of motion), thermal (heat energy), electric, chemical, nuclear, and mechanical energies. All, however, are associated with motion, can be converted from one form to another, and abide by the first law of thermodynamics, or “conservation of energy.”

2 Einstein’s STR proposes the inarguably most recognized equation of modern times (E = MC^2^), or energy equals mass times the square speed of light in a vacuum. His GTR, likewise, despite its obvious complexity, equates stress-energy tensor (left side) with the curvature of space time (right side), that is (in its simple form) 8πT = G. Put differently, space-time curvature possesses its own gravitational self-energy.

3 Force (as well as momentum) possess a vector quantity, indicating it has both magnitude and direction, unlike energy (or its equivalent, mass) which is regarded as being a scalar, and therefore possessing magnitude alone. Four forces of nature are recognized in physics and they include: electrodynamic (i.e., influences electrically charged particles, such as attraction between the positively charged proton and the negatively charged electron), strong (i.e., acts on subatomic scale; influences and binds together quarks within protons and neutrons), weak (i.e., acts on subatomic scale; responsible for radioactive decay, as when one particle decays into another), and gravitation (i.e., reflects the long-distance attraction between two, or more, objects possessing mass or energy).

4 Realistically, both healthy life situations and illness experiences can have positive and negative valences, for example, when illness is sought as a refuge from daily responsibilities and health is avoided because of these unwelcomed responsibilities.

5 The Outcomes Measurement Information System (PROMIS), is a related instrument, developed to obtain self-reported health-related quality of life (HRQOL)? data from sociodemographic-diverse groups of people with a wide range of CIDs. Of the primary summary measures of physical and psychological well-being, the former provides data on fatigue, vitality, vigor and experienced. The latter, relatedly, provides information on respondents’ life-satisfaction, emotional balance, self-concept, autonomy, mastery, optimism and locus of control, all reflective of various facets of PE (Hays et al., 2009; Jacobson et al., 2020).

6 These items were selected, in a slightly revised format, From the Coping Responses Inventory (Moos, 1993) to represent approach (mostly effective, adaptive) coping responses and avoidance (mostly ineffective, non-adaptive) coping responses, respectively.

7 Time could also be considered as an ingredient of such an analysis, although less persuasively, ranging from (a) past-oriented (i.e., energy mostly emanates from, or is stored in, past events, experiences and crises/traumas); (b) present-oriented (i.e., energy is available for current activities and needs); and (c) future-oriented (i.e., energy is anticipated to be freed for future goals and long-term pursuits).
